# Genetic sex validation for sample tracking in next-generation sequencing clinical testing

**DOI:** 10.1186/s13104-024-06723-w

**Published:** 2024-03-03

**Authors:** Jianhong Hu, Viktoriya Korchina, Hana Zouk, Maegan V. Harden, David Murdock, Alyssa Macbeth, Steven M. Harrison, Niall Lennon, Christie Kovar, Adithya Balasubramanian, Lan Zhang, Gauthami Chandanavelli, Divya Pasham, Robb Rowley, Ken Wiley, Maureen E. Smith, Adam Gordon, Gail P. Jarvik, Patrick Sleiman, Melissa A. Kelly, Harris T. Bland, Mullai Murugan, Eric Venner, Eric Boerwinkle, the eMERGE III consortium, Debra J. Abrams, Samuel E. Adunyah, Ladia H. Albertson-Junkans, Berta Almoguera, Paul S. Appelbaum, Samuel Aronson, Sharon Aufox, Lawrence J. Babb, Hana Bangash, Melissa A. Basford, Meckenzie Behr, Barbara Benoit, Elizabeth J. Bhoj, Sarah T. Bland, Kenneth M. Borthwick, Erwin P. Bottinger, Deborah J. Bowen, Mark Bowser, Murray Brilliant, Adam H. Buchanan, Andrew Cagan, Pedro J. Caraballo, David J. Carey, David S. Carrell, Victor M. Castro, Rex L. Chisholm, Wendy Chung, Christopher G. Chute, Brittany B. City, Ellen Wright Clayton, Beth L. Cobb, John J. Connolly, Paul K. Crane, Katherine D. Crew, David R. Crosslin, Renata P. da Silva, Jyoti G. Dayal, Mariza De Andrade, Josh C. Denny, Ozan Dikilitas, Alanna J. DiVietro, Kevin R. Dufendach, Todd L. Edwards, Christine Eng, David Fasel, Alex Fedotov, Stephanie M. Fullerton, Birgit Funke, Stacey Gabriel, Vivian S. Gainer, Ali Gharavi, Joe T. Glessner, Jessica M. Goehringer, Adam S. Gordon, Chet Graham, Heather S. Hain, Hakon Hakonarson, John Harley, Margaret Harr, Andrea L. Hartzler, Scott Hebbring, Jacklyn N. Hellwege, Nora B. Henrikson, Christin Hoell, Ingrid Holm, George Hripcsak, Alexander L. Hsieh, Elizabeth D. Hynes, Darren K. Johnson, Laney K. Jones, Yoonjung Y. Joo, Sheethal Jose, Navya Shilpa Josyula, Anne E. Justice, Elizabeth W. Karlson, Kenneth M. Kaufman, Jacob M. Keaton, Eimear E. Kenny, Dustin L. Key, Atlas Khan, H. Lester Kirchner, Krzysztof Kiryluk, Terrie Kitchner, Barbara J. Klanderman, David C. Kochan, Emily Kudalkar, Benjamin R. Kuhn, Iftikhar J. Kullo, Philip Lammers, Eric B. Larson, Matthew S. Lebo, Ming Ta Michael Lee, Kathleen A. Leppig, Chiao-Feng Lin, Jodell E. Linder, Noralane M. Lindor, Todd Lingren, Cong Liu, Yuan Luo, John Lynch, Bradley A. Malin, Brandy M. Mapes, Maddalena Marasa, Keith Marsolo, Elizabeth McNally, Frank D. Mentch, Erin M. Miller, Hila Milo Rasouly, Shawn N. Murphy, Melanie F. Myers, Bahram Namjou, Addie I. Nesbitt, Jordan Nestor, Yizhao Ni, Janet E. Olson, Aniwaa Owusu Obeng, Jennifer A. Pacheco, Joel E. Pacyna, Thomas N. Person, Josh F. Peterson, Lynn Petukhova, Cassandra Pisieczko, Siddharth Pratap, Megan J. Puckelwartz, Alanna K. Rahm, James D. Ralston, Arvind Ramaprasan, Luke V. Rasmussen, Laura J. Rasmussen-Torvik, Dan M. Roden, Elisabeth A. Rosenthal, Maya S. Safarova, Avni Santani, Juliann M. Savatt, Daniel J. Schaid, Steven Scherer, Baergen I. Schultz, Aaron Scrol, Soumitra Sengupta, Gabriel Q. Shaibi, Ning Shang, Himanshu Sharma, Richard R. Sharp, Yufeng Shen, Rajbir Singh, Jordan W. Smoller, Duane T. Smoot, Ian B. Stanaway, Justin Starren, Timoethia M. Stone, Amy C. Sturm, Agnes S. Sundaresan, Peter Tarczy-Hornoch, Casey Overby Taylor, Lifeng Tian, Sara L. Van Driest, Matthew Varugheese, Lyam Vazquez, David L. Veenstra, Digna R. Velez Edwards, Miguel Verbitsky, Kimberly Walker, Nephi Walton, Theresa Walunas, Firas H. Wehbe, Wei-Qi Wei, Scott T. Weiss, Quinn S. Wells, Chunhua Weng, Marc S. Williams, Janet Williams, Leora Witkowski, Laura Allison B. Woods, Julia Wynn, Yanfei Zhang, Jodell Jackson, Cynthia Prows, Lisa Mahanta, Heidi L. Rehm, Richard A. Gibbs, Donna M. Muzny

**Affiliations:** 1 Baylor College of Medicine, Human Genome Sequencing Center (HGSC), Houston, TX USA; 2 Department of Molecular and Human Genetics, Baylor College of Medicine, Houston, TX USA; 3 Laboratory for Molecular Medicine (LMM), Mass General Brigham, Cambridge, MA USA; 4 Department of Pathology, Massachusetts General Hospital, Harvard Medical School, Boston, MA USA; 5 Broad Institute of MIT and Harvard, Cambridge, MA USA; 6 Division of Genomic Medicine, National Human Genome Research Institute, Bethesda, MD USA; 7 Center for Genetic Medicine, Northwestern University Feinberg School of Medicine, Chicago, IL USA; 8 Division of Medical Genetics, Department of Medicine, University of Washington Medical Center, Seattle, WA USA; 9 Center for Applied Genomics, Children’s Hospital of Philadelphia, Philadelphia, PA USA; 10 Genomic Medicine Institute, Geisinger, Danville, PA USA; 11 Vanderbilt University Medical Center, Nashville, TN USA; 12 Human Genetics Center, University of Texas Health Science Center at Houston, Houston, TX USA; 13 Division of Human Genetics, Cincinnati Children’s Hospital Medical Center, Cincinnati, OH USA

**Keywords:** Next-generation sequencing (NGS), Clinical testing, Sex concordance, SNP genotyping

## Abstract

**Objective:**

Data from DNA genotyping via a 96-SNP panel in a study of 25,015 clinical samples were utilized for quality control and tracking of sample identity in a clinical sequencing network. The study aimed to demonstrate the value of both the precise SNP tracking and the utility of the panel for predicting the sex-by-genotype of the participants, to identify possible sample mix-ups.

**Results:**

Precise SNP tracking showed no sample swap errors within the clinical testing laboratories. In contrast, when comparing predicted sex-by-genotype to the provided sex on the test requisition, we identified 110 inconsistencies from 25,015 clinical samples (0.44%), that had occurred during sample collection or accessioning. The genetic sex predictions were confirmed using additional SNP sites in the sequencing data or high-density genotyping arrays. It was determined that discrepancies resulted from clerical errors (49.09%), samples from transgender participants (3.64%) and stem cell or bone marrow transplant patients (7.27%) along with undetermined sample mix-ups (40%) for which sample swaps occurred prior to arrival at genome centers, however the exact cause of the events at the sampling sites resulting in the mix-ups were not able to be determined.

**Supplementary Information:**

The online version contains supplementary material available at 10.1186/s13104-024-06723-w.

## Introduction

The implementation of next-generation sequencing (NGS) technologies in clinical laboratories [1–4] typically involves three phases: (i) the pre-analytic phase including sample collection, DNA extraction and shipment; (ii) the analytic phase of NGS library preparation, DNA sequencing, bioinformatics analysis; and (iii) a post-analytic phase including clinical report generation and delivery. Each phase is inherently subject to sample tracking and identification errors, with prior reports of more than 46% of errors occurring during the pre-analytical phase, caused by inappropriate test requests, order entry errors, patient misidentification, and labelling errors [5]. Validation and tracking of sample identity therefore is a basic and important aspect of effective clinical NGS testing.

DNA-based methods for sample tracking include genotyping of short tandem repeats (STRs) or single nucleotide polymorphisms (SNPs) [6–8]. STRs are generally located in non-coding regions, prone to high sequencing error rates, and often require longer than typical sequencing read lengths to precisely define the number of repeats, limiting their application. In contrast, SNPs are ubiquitous in the genome and simple to assay [9–11]. In this study, a 96-SNP panel was used to track samples through the clinical NGS workflow in the National Institute of Health’s Electronic Medical Records and Genomics Phase III (eMERGE) program [12]. The network linked together 11 sample collection sites and 2 clinical genetic testing laboratories, the Human Genome Sequencing Center Clinical Laboratory at Baylor College of Medicine (BCM-HGSC-CL) and the Mass General Brigham Laboratory for Molecular Medicine (LMM) in partnership with the Clinical Research Sequencing Platform (CRSP) at the Broad Institute of MIT and Harvard. A total of 25,015 clinical DNA samples were processed. The 96-SNP panel-based procedure provided a robust method for sample tracking in the clinical NGS workflow and showed that the testing of sex can provide a valuable quality control tool.

## Methods

### Fluidigm SNP genotyping assay

Two clinical laboratories harmonized methods for the program [12] and utilized a 96-SNP panel but incorporated different selected SNPs to track samples and determine ancestry. Each 96-SNP panel contained one subset of SNPs on the sex-chromosomes. The autosome SNPs are within the target region of the capture design used in the eMERGE program (Additional files 1, 2) [12]. Assays were performed according to the manufacturer’s recommendations.

The BCM-HGSC-CL’s 96-SNP panel replaced 19 of the original Fluidigm SNPtrace 96 sites to match genomic regions specifically targeted in eMERGE III. The remaining sites included 3 SNPs on Chromosome X and 3 on Chromosome Y [13, 14]. At the Broad Institute, the chosen SNPs included 95 autosomal SNPs and 1 sex determining assay locus, covering the AMELX and AMELY gene (AMG_3B) with a sex-specific 6 base-pair insertion/deletion.

### Illumina Infinium HumanCoreExome SNP array assays and NGS

The HumanCoreExome v1-3 BeadChips contain 500K variant sites, including more than 12,900 located on the X chromosome, that are informative for genetic sex prediction. Infinium SNP array assay were performed with 200 ng genomic DNA according to manufacturer’s instructions. DNA sequencing for the eMERGE phase III program has been described previously [12].

## Results

**Fig. 1:**
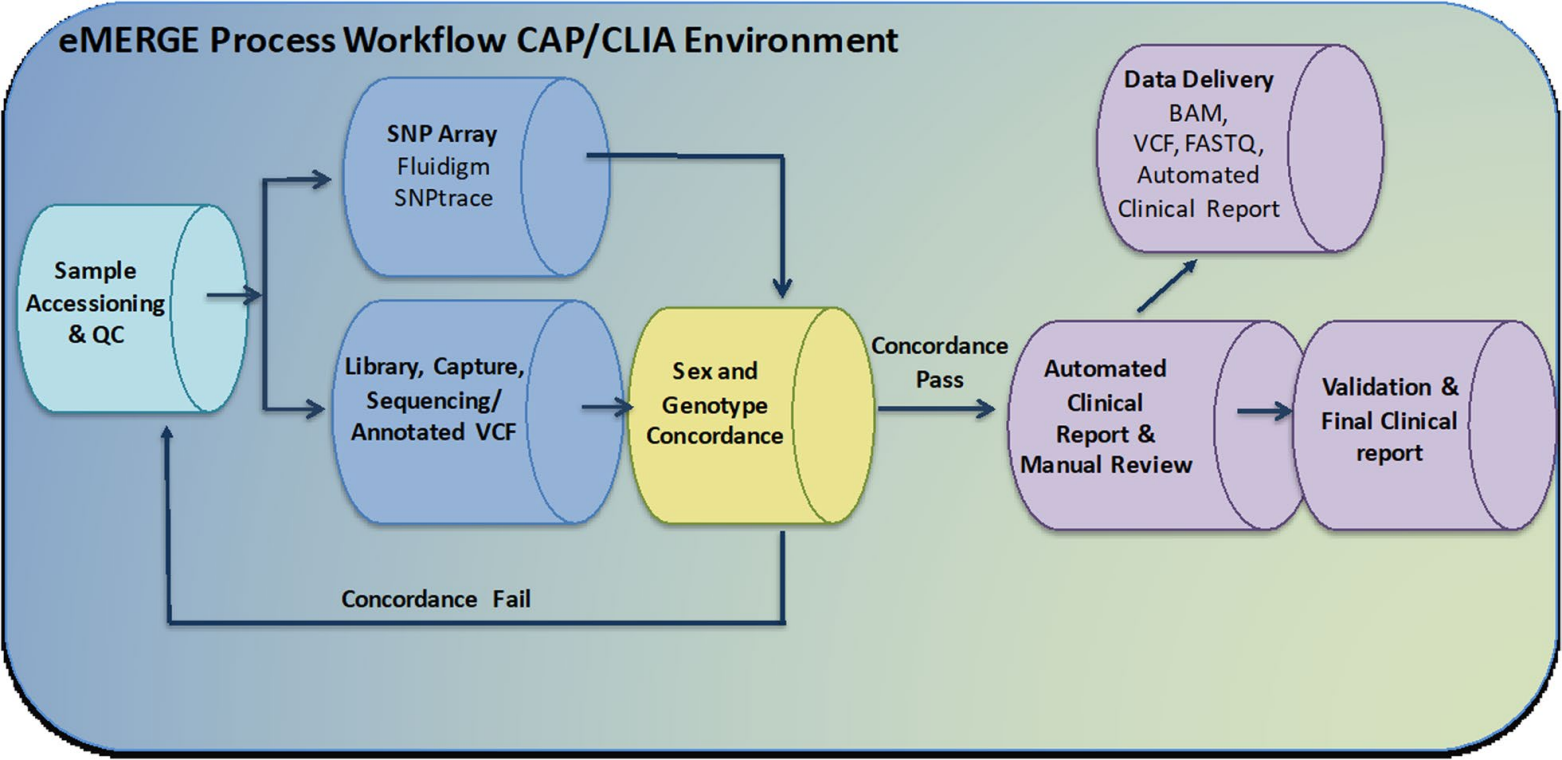
eMERGE sample processing workflow. Steps indicating where aliquots of DNA are taken from samples that are presented to the Clinical DNA Sequencing Laboratory for accession, to test via the Fluidigm 96-SNP panel assay. Data from the Fluidigm 96-SNP panel assay are compared with DNA sequence data from the DNA sequencing pipeline as a quality control step, ahead of the Automated Clinical Reporting step

As a first step towards assessing sample swaps during the analytic phase in NGS testing, we tested the concordance between data generated from the 96-SNP panel genotyping and the DNA sequence data at each of the two Genome Characterization Centers. The BCM-HGSC-CL and LMM/Broad laboratories utilized the same analytical platform foundation, employing slightly different SNP sites for the assays, but generally similar workflows (Fig. 1). The average SNP call rates were 97.3% and 97.5% for the 25,015 samples processed at the BCM-HGSC-CL and the LMM/Broad, respectively. No sample swaps were identified during the analytic NGS testing phase. Next, we compared the 96-SNP panel genotype-based sex to reported sex at the time of sample accessioning, where a total of 110 (0.44%) non-concordant cases from two testing laboratories were identified. The two testing laboratories utilized slightly different workflows to technically validate the sex discrepancies.

**Fig. 2:**
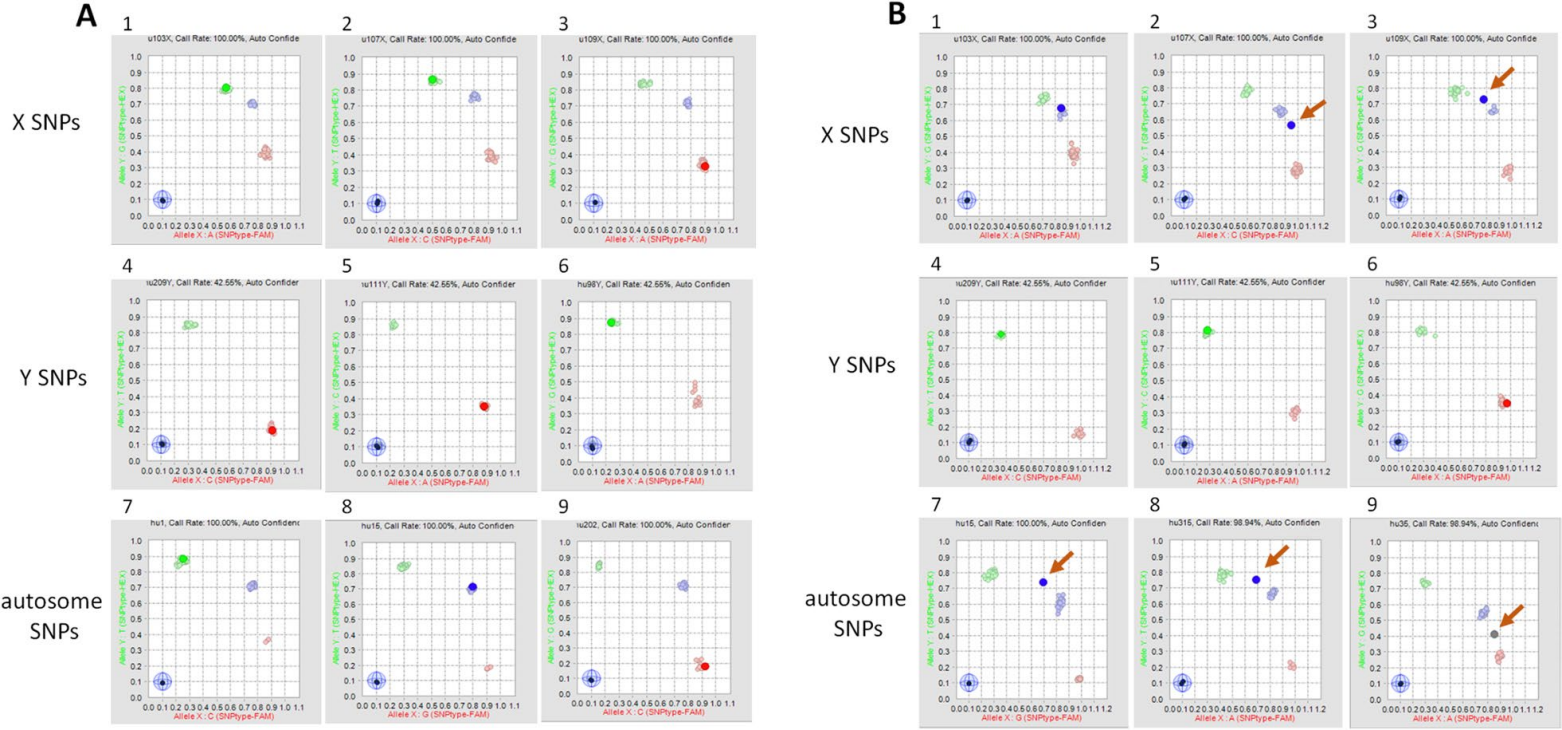
Scatter plot analysis of 96-SNP panel reveals sample contamination. Scatter plot analysis from vendor software, showing a normal DNA male sample (**A**) or a contaminated sample containing a mixture of male and female DNAs (**B**). Panels 1–3 SNPs on X chromosome; panels 4–6 SNPs on Y chromosome; panels 7–9 autosomal SNPs. Each panel shows the data from a single SNP, as compared to clusters from all other SNPs. Clusters are shown as either homozygous (red or green), or heterozygous (blue) positions. In panels B2, 3, 7–9 single SNPS are represented as outside the expected (arrows) resulting in erroneous or ‘no-call’ from the software

At the BCM-HGSC-CL, of the 14,515 samples processed, 73 samples with sex discrepancies were re-tested with the same 96-SNP panel. Identical results were obtained for 70 of the re-tested samples (Table 1). For the remaining 3 cases, where the sex provided on test requisition was male, non-concordant or ambiguous data were observed between the initial and the repeated assays. For two of these samples, the automated software calls from one of each duplicate assays indicated that the DNA source was from individuals with Klinefelter Syndrome (47, XXY). However, further review of the SNP scatter plots for autosome and sex SNPs indicated that the inconsistent sex calls most likely resulted from sample contamination involving a mixture of male and female DNAs (Fig. 2). The third sample was called as female with lower confidence initially. In the repeated assay, one of the X SNPs failed to call due to localization in between clusters in plot analysis. This is most likely due to the female sample mixed up with some DNA sample from another female.

**Table 1:** Comparison of genetic sex determined in various assays and reported sex on test requisition

Sequencing site	Total	Sample providing site	Sex on test requisition	Sex from 1st Fluidigm array	Sex from 2nd Fluidigm array	Sex from Illumina array	Sex from sequencing data	Sample number
BCM-HGSC-CL	73	Site 1	Male	Female	Female	Female	–	5
			Female	Male	Male	Male	–	5
		Site 2	Male	Female	Female	Female	–	13
			Male	Female	Klinefelter	Female	–	1
			Female	Male	Male	Male	–	7
		Site 3	Male	Female	Female	Female	–	3
			Female	Male	Male	Male	–	3
			Female	Male	Male	NA^a^	–	1
		Site 4	Male	Female	Female	Female	–	7
			Female	Male	Male	Male	–	9
			Male	Klinefelter	Female	NA^a^	–	1
		Site 5	Male	Female	Female	Female	–	6
			Male	Female	No Call	Female	–	1
			Female	Male	Male	Male	–	4
		Site 6	Male	Female	Female	Female	–	4
			Female	Male	Male	Male	–	3
LMM/broad	37	Site 7	NA^b^	Female	–	–	Female	1
			Female	NA^c^	–	–	Male	1
			Male	Female	–	–	Female	16
			Female	Male	–	–	Male	13
		Site 8	Male	Female	–	–	Female	1
		Site 9	Male	Female	–	–	Female	1
			Female	Male	–	–	Male	2
		Site 10	Male	Female	–	–	Female	1
			Female	Male	–	–	Male	1

^a^Insufficient gDNA for Illumina array

^b^Sex not reported on requisition form

^c^Sex not called in assay; *NA* not available

Next, Illumina HumanCore Exome Arrays were utilized as an orthogonal high-density hybridization genotyping assay to further test 71 of the 73 samples with sex inconsistencies except two samples which had insufficient genomic DNA (Table 1). HumanCore Exome Array results confirmed 96-SNP panel genotyping sex data, including the suspected two contaminated female samples with additional male or other female DNA.

At the Broad/LMM, the reported sex from the test requisition was compared with the genetic sex determined by both the Fludigm genotyping assay and the data from the eMERGE III sequencing panel. Of the 10,500 samples processed, 151 were initially either identified as discordant or had no sex determination. For 95 samples, the Fluidigm assay data could not return a sex determination, however the sequencing sex matched the reported sex for each and no further action was taken. For 19 of the remaining 56 samples, the sequencing and reported sex were concordant, but did not match the genotyping determined sex. Further review of these 19 samples showed that the genotyping assay calls were generally borderline or low confidence calls, suggesting sub-optimal performance of the single sex determining SNP as the reason for the data discrepancy, rather than either a sex reporting error at accession or sample mix-up in the testing laboratory. The remaining 37 samples had highly confident sex determination calls from both the SNP assay and the subsequent DNA sequencing that were concordant, but did not match the site reported sex (Table 1).

Internal tracking showed that none of the 110 confidently identified sex discrepant samples occur within the clinical DNA sequencing laboratories and that most errors were likely introduced prior to shipment of samples. Sampling sites identified handling errors from test requisitions, sample extraction, and sample handling procedures for 54 cases. Forty-six of these had information that was incorrectly or incompletely entered on the test requisitions and were resolved by examination of other records. In 6 other cases, it was determined that incorrect samples had been shipped from the sampling sites to the genome centers. Biological explanations for the discrepant tracking data were identified for an additional 12 cases. In 4 of these 12 cases, further examination of records revealed that the samples were provided by transgender participants. In addition, 8 sex discrepant samples were determined to be from individuals who had received stem cell or bone marrow transplants. Causes of the sample genetic vs. reported sex discrepancy are listed in Table 2.

**Table 2:** Causes of sample sex discrepancy

Sex discrepant categories	BCM-HGSC-CL samples	LMM/broad samples	Total
Sampling site errors			
Incorrect/incomplete information on test requisition	45	1	46
Error during DNA extraction	0	2	2
Incorrect sample shipped	6	0	6
Transgender	2	2	4
Stem cell/bone marrow transplant recipient	8	0	8
Not solved/under investigation	12	32	44
**Total sex discrepancies**	**73**	**37**	**110**

Where possible, the information on test requisition forms was amended and correct clinical reports were issued for 45 cases processed at the BCM-HGSC-CL, or the incorrect samples were replaced and re-processed. Twelve cases sequenced at the BCM-HGSC-CL with sample mix-ups due to unknown causes were withdrawn from the study. Similarly, 32 unsolved cases sequenced at LMM/Broad were either withdrawn or remained under investigation.

## Discussion

To identify sample swaps during the processing of 25,015 clinical samples in the NIH eMERGE III program, two clinical DNA sequencing laboratories first utilized a Fluidigm-based 96-SNP panel assay to track internal processes. These analyses indicated no sample swaps had occurred in the time interval between sample arrival at the testing laboratories and the delivery of the final DNA sequencing data. In contrast, when the test was expanded to predict the concordance between the self-reported sex of participants at the time of their initial enrollment, with a predicted sex-by-genotype, there were 110 discordant samples. A battery of follow-up tests indicated that these likely arose before the materials were received at the clinical DNA sequencing laboratories. The bases of the sample tracking errors at sample collection sites were determined in 66 of the 110 cases (60%), while leaving the remaining 44 cases unsolved and under investigation. Of these 66 resolved cases, the largest source for the initial discordance occurring in 54 cases (81%) arose from clerical or shipping errors. The remaining 12 cases (18% of the 66 solved) had biological underpinnings that explained the discordant results, as 8 were due to stem cell/bone marrow transplants while 4 were from transgender individuals. Future sample collecting procedures could be modified by including more informative test requisition options to ensure that participants are invited to note these types of events at the time of collection, so that this information is available for quality control.

The 96-SNP panel has proven value for precise sample tracking [15]. In general, 20 informative SNP loci are sufficient for unique individual sample identification [16, 17]. Other SNP panels have been used for identification of human samples [9, 18, 19]. A low-density QC genotyping array launched by Illumina which includes 15,949 markers has been utilized in genomic-based clinical diagnostics [20]. Our studies showed that these two different SNP platforms exhibited consistent results when applied for sex identification. In comparison to the use of the Illumina Infinium array platform, the workflow for the 96-SNP panel assay is faster (1-day workflow vs 3-day workflow) and more cost-effective (chip price for SNPtrace is about 15% of HumanCoreExome Array per sample). However, the Illumina Infinium array platform provides more information on linkage analysis, HLA haplotyping, ethnicity determination and other genetic information in addition to fingerprinting and thus may be preferred in some scenarios. It may also take into account the sex prediction accuracy of the two methods, the error rate, albeit low, as well as the cost of re-testing that may be necessary in some cases due to low data quality. Other commercial systems are also available to substitute for the platforms described here if they provide cost-effective and precise data with similar qualities.

This level of tracking error is unacceptable for ongoing clinical practice, but the study does not represent the levels that will be expected in further clinical programs. At least one laboratory declared their initial sample enrollments as ‘research samples’ and thus committed to later repeat assays under a fully compliant protocol, to verify any findings that may impact care. Others were able to quickly identify points of error and rectify their protocols to ensure faithful future sample handling. All sites committed to rechecking of records and reconciling actionable findings with orthogonal data, including family histories and biochemical tests, before returning results. The ‘lessons learned’ from these analyses ensure that a repeat of the same program would likely minimize any similar errors.

## Limitations

While false positive rates are low for this application of SNP trace, false negative rates will be high. Here, the overall level of genetic and reported sex discordance of 0.44% is likely an underestimate of the true error rate in this study, as the misclassification of genetic sex from a random sample swap would be expected to result in incorrect, erroneous assignment, only 50% of the time. The true ratio may be skewed by factors introducing a sex-bias in the direction of misclassification. This could be caused by skewed phenotypes of individuals with sex chromosome anomalies or that gender obfuscation may be socially driven in an unequal manner, depending on the gender identity of the individual. Overall, the rate is likely higher than the 0.44% identified here, but not anticipated to be higher than twice that level.

### Supplementary Information

**Additional file 1: Table S1.** 96-SNP panel design—BCM-HGSC-CL.

**Additional file 2: Table S2.** List of 96 SNPs in LMM/broad (CRSP) PCR panel design.

## Data Availability

Data are available in dbGaP for controlled public access (phs001616.v1.p1).

## Abbreviations

HGSC: Human Genome Sequencing Center
LMM: Laboratory for Molecular Medicine
NGS: Next-generation DNA sequencing
STR: Short tandem repeat
SNP: Single nucleotide polymorphism
PCR: Polymerase chain reaction
eMERGE: Electronic Medical Records and Genomics
EMR: Electronic medical record
HGSC-CL: Human Genome Sequencing Center Clinical Laboratory
BCM: Baylor College of Medicine
CRSP: Clinical Research Sequencing Platform
NHGRI: National Human Genome Research Institute
IRB: Institutional review board
